# Clinical and Laboratory Characteristics of Symptomatic and Asymptomatic Neurosyphilis in HIV-Negative Patients: A Retrospective Study of 264 Cases

**DOI:** 10.1155/2019/2426313

**Published:** 2019-05-06

**Authors:** Wurong Li, Meijuan Jiang, Dongmei Xu, Cheng Kou, Lei Zhang, Junhua Gao, Kaiyu Qin, Wenqing Wu, Xinghu Zhang

**Affiliations:** ^1^Department of Neurology, Beijing Tiantan Hospital, Capital Medical University, Beijing 100069, China; ^2^China National Clinical Research Center for Neurological Diseases, Beijing 100069, China; ^3^Department of Neurology, Beijing Ditan Hospital, Capital Medical University, Beijing 100015, China

## Abstract

A retrospective study was performed to compare the differences in clinical and laboratory features of asymptomatic neurosyphilis (ANS) and symptomatic neurosyphilis (SNS). A total of 264 HIV-negative inpatients with neurosyphilis were enrolled from Beijing Ditan Hospital and Beijing Tiantan Hospital between January 2014 and May 2018, including 110 SNS and 154 ANS. The SNS group had more patients in males, older median age and without antisyphilis treatment than ANS group (P<0.001, P<0.001, and P<0.001, respectively). The laboratory findings showed that the SNS group had higher pretreatment serum rapid plasma regain (RPR) titer, current serum RPR titer, cerebrospinal fluid (CSF) white blood cell (WBC) counts, CSF protein concentrations, and higher positive CSF RPR rate than those in the ANS group (P=0.011, P<0.001, P<0.001, P<0.001, and P<0.001, respectively). The multivariate logistic regression analysis revealed that male (OR=2.833, P=0.009), age≥45 years (OR=3.611, P=0.001), without antisyphilis treatment (OR=0.247, P<0.001), higher current serum RPR titer (OR=1.373, P=0.022), positive CSF RPR (OR=4.616, P<0.001), and higher CSF protein concentration (OR=1.017, P=0.026) were independent risk predictors for SNS. Therefore, clinical and laboratory features between SNS and ANS are quietly different. Male gender, age≥45 years, and lack of antisyphilis treatment are risk factors for SNS. The elevated level of serum RPR titer, CSF protein concentration, and CSF RPR titer may indicate the development of neurosyphilis and the aggravation of neurological symptoms.

## 1. Introduction

Neurosyphilis is a chronic infectious disease of central nervous system (CNS) caused by* Treponema pallidum*, which can occur during any stage of the syphilis and represents approximately 30% of untreated cases of the disease [1]. Asymptomatic neurosyphilis (ANS) occurs early in CNS infection. ANS patients have serologic or clinical evidence of syphilis, or both, with CSF pleocytosis, elevated protein, reactive CSF Venereal Disease Research Laboratory (VDRL) or some combination of these abnormalities [2]. But they are neurologically asymptomatic. ANS patients with persistent infection or without treatment were at risk for progression to symptomatic neurosyphilis (SNS), mainly including symptomatic syphilitic meningitis, meningovascular neurosyphilis, intracranial gummas, general paresis, and tabes dorsalis [3]. Treatment of SNS patients may stop disease progression, but there will be residual stroke symptoms and signs, dementia, or sensory ataxia. Unfortunately, little data on the relationship between ANS and SNS among HIV-negative patients are available. In the present study, we retrospectively analyzed the differences in clinical and laboratory features of 154 cases with ANS and 110 cases with SNS and explored the risk factors of SNS.

## 2. Methods

### 2.1. Study Design

The continuous inpatient electronic medical records at the Department of Neurology, Beijing Ditan Hospital, Capital Medical University, and Department of Neurology, Tiantan Hospital, Capital University, were reviewed for neurosyphilis between January 2014 and May 2018. All patients underwent lumber puncture to rule out neurosyphilis, either because they had neurological and/or ophthalmic symptoms or signs or a serofast status without clinical symptoms or signs. After a detailed evaluation based upon diagnostic criteria, 264 patients with neurosyphilis were included in this study. All of the enrolled patients were HIV negative. This study was approved by the local ethics committee of Beijing Ditan Hospital, Capital Medical University, and written informed consent was obtained from all participants.

### 2.2. Diagnostic Criteria

The diagnosis of neurosyphilis remains challenging as there are no gold standard tests. The diagnosis of neurosyphilis is usually based on clinical and CSF findings. The nontreponemal test that is most commonly used to diagnose neurosyphilis from CSF is the VDRL. Zhu L et al. found that CSF RPR has sensitivities and specificities comparable to those of CSF-VDRL and recommended CSF RPR as an alternative test for neurosyphilis diagnosis in HIV-negative populations [4]. The European also recommend CSF RPR as the diagnosis of neurosyphilis for its high specificity [5]. In contrast, the CSF treponemal antibody tests are more sensitive and relatively nonspecific eliminating neurosyphilis as a diagnostic possibility [6]. In our study, the CSF RPR and the CSF treponema pallidum particle agglutination (TPPA)/fluorescent treponemal antibody absorption (FTA-ABS) were used for the diagnosis of neurosyphilis. The diagnostic criteria for neurosyphilis were based on the guidelines of CDC in the USA and Europe and related literatures [4–7]. The neurosyphilis cases were subdivided into confirmed neurosyphilis and presumptive neurosyphilis. Confirmed neurosyphilis is defined as syphilis of any stage with positive RPR in CSF. Presumptive neurosyphilis is defined as syphilis of any stage with a negative CSF RPR test and both of the following: (1) positive CSF TPPA/ FTA-ABS, and (2) elevated CSF WBC count (>5/ul) or elevated CSF protein concentration (>45mg/dl) in the absence of other known causes of these abnormalities.

The enrolled patients were divided into ANS group and SNS group according to the clinical manifestations. ANS was defined as syphilis of any stage that met the laboratory criteria for neurosyphilis but without clinical symptoms or signs. SNS was defined as syphilis of any stage that met the laboratory criteria for neurosyphilis without other known causes for their clinical symptoms or signs.

### 2.3. Statistical Analysis

Data was analyzed by IBM SPSS 25.0 version (SPSS Inc., IL, USA). Continuous variables were described using median and interquartile range (IQR), while categorical variables were described by numbers and percentages. Two group patients were compared using the independent two-sample *t* test or Mann–Whitney *U* test. Associations between categorical variables were assessed by the chi-square test. Univariate analysis was utilized to determine the factors associated with SNS. The odds ratio (OR) was estimated with 95% confidence intervals (CIs) from the bivariate analysis, and factors with P<0.1 were further identified in the multivariate logistic analysis. All statistical assessments were two tailed, and P<0.05 was considered statistically significant. The RPR titer measured by the double dilution method was applied to analyze after a log transformation (log_2_ 1/RPR titer).

## 3. Results

### 3.1. General Characteristics of Study Patients

A total of 264 neurosyphilis patients were enrolled in the study. The baseline characteristics are shown in Table 1. There were 160 male and 104 female patients, respectively. The median age was 46 (IQR, 34-55) years.

A total of 154 (58.3%) patients were diagnosed as ANS. The median age of these patients was 39 (IQR, 30~52) years, and the patients included 72 males and 82 females. Of these patients, 99 cases received benzathine penicillin G treatment prior to the diagnosis of neurosyphilis, while 55 cases had no treatment history. Before antisyphilis treatment, 157 patients had available baseline serum RPR titer (median titer, 1:32; IQR, 1:8~1:64). All patients had current serum RPR titer (median titer, 1:8; IQR, 1:8~1:32) (when lumbar puncture was performed). 45 patients had positive CSF RPR, of which 19 had RPR 1:1, 21 had RPR 1:2, 3 had RPR 1:4, 1 had RPR 1:8, and 1 had RPR 1:32. A total of 125 patients had CSF WBC>5/ul; the median was 7(IQR, 5~11) /ul. 44 patients had CSF protein concentration>45mg/dl; the median was 35.3 (IQR, 26.2~46.6) mg/dl. All patients had positive TPPA and FTA-ABS-IgG in serum and CSF. Only 33 patients had seropositive FTA -ABS-IgM and 1 patient had positive CSF FTA-ABS- IgM.

A total of 110 (41.7%) patients were diagnosed as SNS. The median age of these patients was 50 (IQR, 44-58) years, and the patients included 88 males and 22 females. Of these patients, only 43 cases had benzathine penicillin G treatment prior to the diagnosis of neurosyphilis. The most common type of SNS was general paresis (51 cases), followed by meningovascular neurosyphilis (23 patients), tabes dorsalis (14 patients), syphilitic meningitis (13 patients), syphilitic optic atrophy (6 cases), and syphilitic myelitis (3 patients). A total of 99 patients had available pretreatment RPR titer (median titer, 1:32; IQR, 1:16~1:64). All patients had current serum RPR titer (median titer, 1:32; IQR, 1:8~1:64). 90 patients had positive CSF RPR titer, of which 24 had RPR 1:1, 24 had RPR 1:2, 22 had RPR 1:4, 13 had RPR 1:8, 6 had RPR 1:16, and 1 had RPR 1:32. 91 patients had CSF WBC>5/ul, the median was 10 (IQR, 6~33) /ul. 75 patients had CSF protein concentration>45mg/dl; the median was 56.2 (IQR, 43.1~76.8) mg/dl. All patients had positive TPPA and FTA-ABS-IgG in serum and CSF. 35 patients had positive serum FTA-ABS-IgM and 1 patient had positive CSF FTA-ABS-IgM.

### 3.2. Comparison of Clinical and Laboratory Findings between SNS and ANS Group

By comparing the clinical features between ANS and SNS group, it was found that there was a significantly higher proportion of males in the SNS group (80.0%, 88/110) than in the ANS group (46.8%, 72/154) (P<0.001). The age was older in SNS group (median age, 50 years; IQR, 44-58 years) than in ANS group (median age, 39 years; IQR, 30-52 years) (P<0.001). In addition, there were more patient lack of antisyphilis treatment in SNS group (60.9%, 67/110) than in ANS group (35.7%, 55/154) (P<0.001) (Table 1).

As for laboratory findings between the two group, it showed that the pretreatment RPR titer and current RPR titer in SNS group were significantly higher than in ANS group (P=0.011 and P<0.001), but no significant difference of positive FTA-ABS-IgM rates was found in two groups (P=0.052). CSF testing for syphilis found that the incidence of positive RPR rate was significantly higher in the SNS group than in the ANS group (P<0.001). CSF WBC count, CSF protein concentration, and abnormal CSF protein concentration rate were significantly higher in SNS group than those in ANS group (P<0.001, P<0.001, and P<0.001, respectively) (Table 1).

### 3.3. Logistic Regression Analysis

Using Bivariate analysis, it showed that the following factors were more likely related to develop SNS: male (OR=4.556; p<0.001], age≥45 years (OR=3.928; P<0.001), without syphilis treatment (OR=0.357; P<0.001), high pretreatment serum RPR titer (OR=1.246; P=0.008), high current RPR titer (OR=1.579; P<0.001), high CSF WBC (OR=1.012; P=0.005), high CSF protein concentration (OR=1.032; P<0.001), and positive CSF RPR (OR=9.208; P<0.001) (Table 2). In multivariable logistic regression, SNS remained significantly more common related to the following factors: aged≥45 years (OR=3.611; P=0.001), male (OR=2.833; P=0.009), without syphilis treatment (OR=0.247; P<0.001), high current serum RPR titer (OR=1.373; P=0.022), high CSF protein concentration (OR=1.017; P=0.026), and positive CSF RPR (OR=4.616; P<0.001) (Table 2).

## 4. Discussion

ANS occurs early in syphilis infection. ANS patients easily develop subsequent SNS. About 35% of ANS patients developed SNS in natural progression [3, 8]. Hence, clinicians should pay much attention to the related risk factors. Several previous studies concerned with risk factors of neurosyphilis in syphilis individuals found that neurosyphilis was more prevalent in male, higher serum RPR titers, and HIV infection [9–12]. However, the risk factors associated with the development of SNS are still unclear. Besides male gender and higher RPR titers, this study also found that age≥45 years, without antisyphilis treatment, positive CSF RPR and high CSF protein concentration may be associated with the development of SNS.

Benzathine penicillin G treatment can effectively kill Treponema pallidum, block the natural process of syphilis, and naturally reduce the incidence of neurosyphilis. Choe et al. examined the CSF in 70 untreated latent syphilis patients and found 57(81%) patients with CSF abnormalities [13]. Our study showed that 67 (60.9%) SNS patients and 55 (35.7%) ANS patients lacked benzathine penicillin G treatment in their early stage of syphilis infection. Multivariate analysis revealed that lack of benzathine penicillin G treatment was related to develop SNS (OR=0.247, P<0.001), indicating that benzathine penicillin G treatment in the early stage of syphilis may reduce the risk of developing SNS. In the present study, we observed the median current serum RPR titer was significantly lower than pretreatment median serum RPR titer (1:8 vs 1:32) in the ANS group, while there was no change of the titer level (1:32 vs 1:32) in the SNS group. The difference may be related to the fact that more patients in SNS group did not receive benzathine penicillin G treatment. A high serum RPR titer has been reported to be associated with an increased likelihood of neurosyphilis [9]. Further analysis of our study showed that high current RPR titer was an independent risk factor for SNS (OR=1.373, P=0.022). Our results revealed that the RPR titer level is correlated with disease activity, which was consistent with previous studies [9, 14]. Therefore, antisyphilis treatment in the early stage of syphilis is essential to prevent the occurrence and development of neurosyphilis, especially SNS.

In our study, the patients in SNS group were older than those in ANS group. Multivariate analysis revealed that patients older than 45 years was an independent predictor of SNS (OR=3.611, P=0.001). Shi et al. found that the age of 45 years was a correlated risk factor for neurosyphilis in HIV-negative patients [15]. This is probably because the older adults might have a longer duration of syphilis infection. The younger people were more likely to have undergone regular screening tests for syphilis and penicillin treatment, so early diagnosis was possible when they were ANS. There were more males in the SNS group (88/110, 80.0%) than in the ANS group (72/154, 46.8%). Other studies also found that the overall incidence of SNS was higher in males than in females [12, 16, 17]. We speculated the reason for gender-related differences could be that the unhealthy sexual behavior was more common among men.

Previous studies on neurosyphilis were confined to clinical findings, serum RPR titers, and profiles of HIV infection. In our study, we took several CSF parameters into consideration. CSF WBC count had high diagnostic value in ANS and SNS and was highly correlated with the activity of neurosyphilis. Of our patients, we found no difference in proportion of CSF WBC abnormality between the two groups (82.7% vs 81.2%), which was consistent with other studies [18, 19], suggesting that CSF WBC abnormality was in the whole process of neurosyphilis and played a sustained role in neurosyphilis. The CSF WBC counts in the SNS group were significantly higher than that in the ANS group (P<0.001), suggesting that inflammatory activity was more obvious in SNS patients. CSF RPR was also positively correlated with activity of neurosyphilis [5, 7, 20]. This study found more patients in SNS group had positive CSF RPR than that in ANS group (81.8% vs 29.2%). Further analysis showed that positive CSF RPR was an independent risk factor for SNS (OR=4.616 P<0.001), indicating that SNS patients had higher disease activity. There were more patients in ANS group with negative CSF RPR. For diagnosing ANS, it was very important to take the combination of several CSF factors into consideration, including WBC count, protein concentration, and TPPA/FTA-ABS. CSF protein concentration in SNS group was higher than that in ANS group, as well as the proportion of protein abnormality. Further study inferred that high CSF protein concentration was an independent risk factor for SNS (OR=1.017, P=0.026). Therefore, if the CSF protein concentration increases during the follow-up, it may indicate the progress or aggravation of the disease, and timely treatment is necessary.

In theory, the disease duration should also be one of risk factors for SNS, but it is difficult for the patients observed in this study especially those without a history of treatment, to give the exact time of syphilis infection. Therefore, the disease duration is not included in this study.

In conclusion, clinical and lab findings in ANS and SNS groups are quietly different. Male gender, age≥45 years, and lack of antisyphilis treatment are related risk factors for SNS. During the treatment and follow-up of syphilis, the increase of RPR titer level may mean the occurrence and development of neurosyphilis. In addition, the elevated level of CSF RPR titer and CSF protein concentration may indicate the development of neurosyphilis and the aggravation of neurological symptoms.

## Figures and Tables

**Table 1:** Patient Demographics and Baseline Characteristics (N = 264).

Characteristic	SNS (110)	ANS (154)	P value
Sex (male: female)	88:22	72:82	P<0.001
Age (years), median year (IQR)	50 (44, 58)	39 (30, 52)	P<0.001
≤ 30	5, 4.5%	42, 27.3%	NA
31~40	13, 11.8%	37, 24.0%	NA
41~50	38, 34.5%	31, 20.1%	NA
51~60	34, 30.9%	33, 21.4%	NA
≥ 61	20, 18.2%	11, 7.1%	NA
Treatment history (yes: no)	43:67	99:55	P<0.001
Pretreatment serum RPR titer, median (IQR)	1:32 (1:16, 1:64)	1:32 (1:8, 1:64)	P=0.011
1:1 (n, %)	1, 0.9%	0, 0%	NA
1:2 (n, %)	0, 0%	4, 2.6%	NA
1:4 (n, %)	2, 1.8%	10, 6.5%	NA
1:8 (n, %)	14, 12.7%	24, 15.6%	NA
1:16 (n, %)	13, 11.8%	29, 18.8%	NA
1:32 (n, %)	32, 29.1%	36, 23.4%	NA
1:64 (n, %)	14, 12.7%	28, 18.2%	NA
1:128 (n, %)	12, 10.9%	10, 6.5%	NA
≥1:256 (n, %)	11, 10.0%	6, 3.9%	NA
Not available (n, %)	11, 10.0%	7, 4.5%	NA
Current serum RPR titer, median (IQR)	1:32 (1:8, 1:64)	1:8 (1:8, 1:32)	P<0.001
1:1	1, 0.9%	0, 0%	NA
1:2	2, 1.8%	9, 5.8%	NA
1:4	1, 0.9%	16, 10.4%	NA
1:8	27, 24.5%	53, 34.4%	NA
1:16	11, 10.0%	30, 19.5%	NA
1:32	27, 24.5%	28, 18.2%	NA
1:64	18, 16.4%	14, 9.1%	NA
1:128	11, 10.0%	1, 0.6%	NA
≥1:256	12, 10.9%	3, 1.9%	NA
Serum TPPA positive (n, %)	110, 100%	154, 100%	NA
Serum FTA-ABS-IgG positive (n, %)	110, 100%	164, 100%	NA
Serum FTA-ABS-IgM positive (n, %)	35, 38.1%	33, 24.1%	P=0.052
CSF RPR titer (positive: negative)	90:20	45:109	P<0.001
1:1	24, 21.8%	19, 12.3%	NA
1:2	24, 21.8%	21, 13.6%	NA
1:4	22, 20.0%	3, 1.9%	NA
1:8	13, 11.8%	1, 0.6%	NA
1:16	6, 5.5%	0, 0%	NA
1:32	1, 0.9%	1, 0.6%	NA
CSF WBC count, median (IQR), /ul	10 (6, 33)	7 (5, 11)	P<0.001
>5/ul (n, %)	91, 82.7%	125, 81.2%	P=0.746
CSF protein concentration, median (IQR), mg/dl	56.2 (43.1, 76.8)	35.3 (26.2, 46.6)	P<0.001
>45mg/dl (n, %)	75, 68.1%	44, 28.6%	P<0.001
CSF TPPA positive (n, %)	110, 100%	154, 100%	NA
CSF FTA-ABS-IgG positive (n, %)	110, 100%	154, 100%	NA
CSF FTA-ABS-IgM positive (n, %)	1, 0.9%	1, 0.6%	NA

Abbreviation: ANS=asymptomatic neurosyphilis; SNS=symptomatic neurosyphilis; RPR = rapid plasma regain; TPPA=Treponema pallidum particle agglutination; CSF=cerebrospinal fluid; WBC=white blood cells; FTA-ABS=fluorescent treponemal antibody absorption; NA=not available

**Table 2:** Univariate and multivariate analysis for clinical and laboratory predictors of SNS patients.

Factors	Univariate		Multivariate	
	OR (95%CI)	P-value	OR (95%CI)	P-value
Sex, male vs female	4.556 (2.591~8.010)	P<0.001	2.833 (1.394~5.880)	P=0.009
Age, ≥45 vs < 45, years	3.928 (2.308~6.684)	P<0.001	3.611 (1.713~7.612)	P=0.001
Treatment history, no vs yes	0.357 (0.215~0.591)	P<0.001	0.247 (0.119~0.512)	P<0.001
Pretreatment serum RPR titer	1.246 (1.060~1.464)	P=0.008	0.837 (0.643~1.090)	P=0.187
Current RPR titer	1.579 (1.337~1.865)	P<0.001	1.373 (1.047~1.800)	P=0.022
Serum FTA-ABS-IgM, + vs -	1.734 (0.994~3.026)	P=0.053	0.957 (0.413~2.215)	P=0.917
CSF protein concentration, mg/dl	1.032 (1.020~1.046)	P<0.001	1.017 (1.002~1.033)	P=0.026
CSF WBC count, /ul	1.012 (1.004~1.021)	P=0.005	1.001 (0.992~1.010)	P=0.816
CSF RPP, + vs -	9.208 (5.215~16.258)	P<0.001	4.616 (2.185~9.752)	P<0.001

Abbreviation: ANS=asymptomatic neurosyphilis; SNS=symptomatic neurosyphilis; RPR = rapid plasma regain; TPPA=Treponema pallidum particle agglutination; CSF=cerebrospinal fluid; WBC=white blood cells; FTA-ABS=fluorescent treponemal antibody absorption; OR=odds ratio; CI=confidence interval;

## Data Availability

The data used to support the findings of this study are available from the corresponding author upon request.
